# Lungworms (*Metastrongylus* spp.) demonstrated in domestic pigs with respiratory disease: was there a clinical relevance?

**DOI:** 10.1186/s40813-022-00258-x

**Published:** 2022-04-12

**Authors:** Per Wallgren, Emelie Pettersson

**Affiliations:** 1 National Veterinary Institute, SVA, 751 89 Uppsala, Sweden; 2 Department of Clinical Sciences, Swedish University of Agricultural Sciences, Box 7054, 750 07 Uppsala, Sweden

**Keywords:** Pig, Outdoor, *Metastrongylus* spp., *Ascaris suum*, *Trichuris suis*, *Mycoplasma hyopneumoniae*, *Pasteurella multocida*, Pneumonia

## Abstract

**Background:**

An outdoor pig herd was affected by severe respiratory disease in one out of three pastures. At necropsy, *Mycoplasma hyopneumoniae* and *Pasteurella multocida* were detected in the lungs, as well as the lung worm *Metastrongylus apri*. The life cycle of *Metastrongylus* spp. includes earthworms as intermediate hosts, and since domestic pigs mainly are reared indoors, lungworm infections have not been diagnosed in domestic pigs in Sweden for decades, not even in pigs reared outdoors. Therefore, this disease outbreak was scrutinised from the view of validating the impact of *Metastrongylus* spp.

**Results:**

At the time of the disease outbreak, neither eggs of *Metastrongylus* spp., *Trichuris suis* nor *Ascaris suum* were detected in faeces of pigs aged ten weeks. In contrast, five-months-old pigs at the pasture with respiratory disease shed up to 3800 eggs per gram (Epg) of *Ascaris suum* and up to 1100 Epg of *Trichuris suis*, whereas eggs of these parasites were not demonstrated in healthy pigs aged six months at another pasture. Low numbers of eggs from *Metastrongylus* spp. (< 150 Epg) were seen in faecal samples from both these age categories. At slaughter, seven weeks later, ten normal weighted pigs in the preceding healthy batch were compared with ten normal weighted and five small pigs from the affected batch. Healing *Mycoplasma*-like pneumonic lesions were seen in all groups. All small pigs shed eggs of *Ascaris suum* in the faeces, compared to around 50% of the larger pigs. *Metastrongylus* spp. were demonstrated in 13 of the 25 pigs (52%), representing all groups included.

**Conclusion:**

As *Metastrongylus* spp. were demonstrated regardless of health status, and also in another healthy outdoor herd, the impact of *Metastrongylus* spp. on the outbreak of respiratory disease was depreciated. Instead, a possible association with a high burden of *Ascaris suum* was suggested to have preceded the severe outbreak with respiratory disease in the affected herd. Further, it was concluded that *Metastrongylus* spp. will escape detection at routine meat inspections made at slaughterhouses, and as they appeared to generally not induce clinical signs of respiratory disease *Metastrongylus* spp. may be more common in outdoor production than previously believed.

## Background

Porcine lungworms (*Metastrongylus* spp.) are nematodes with earthworms as intermediate hosts [1]. Pigs may become infected when they ingest earthworms that contain third stage lungworm larvae. Further development of the lungworm occurs in the pig, and adult lungworms can be found in the bronchi and bronchioles of the lungs. Adult lungworms are thin, but can reach a length of approximately 50 mm. They may cause respiratory illness [1], especially in young individuals where the bronchioles may be obstructed by adult lungworms [2]. Such problems may be enhanced by other concurrent infections [3] and/or by nutritional deficiencies [4]. Lungworms are, however, often neglected as a cause of respiratory diseases in domestic pigs. The reason for this is that pigs are mainly reared indoors where there is no access to the intermediate host [5], and eggs of *Metastrongylus* spp. were not demonstrated in any faecal sample in a recently published Swedish parasite-point-prevalence study [6]. Thus, lungworms are rarely diagnosed in domestic pigs reared indoors, and therefore also rarely discussed in pigs reared outdoors.

During the summer of 2021 a pasture housing a batch of 131 growing pigs outdoors from an approximate mean live weight of 30 kg to around 130 kg was affected by severe respiratory disease. In total, 15 of the pigs died, whereof seven had been unsuccessfully treated with penicillin. However, another 23 affected pigs that had been medicated with penicillin early during the course of disease survived. Two pigs that had died were necropsied and diagnosed with pneumonic lesions resembling those caused by *Mycoplasma hyopneumoniae*. Nevertheless, pigs that were medically treated immediately when clinical signs of respiratory disease were observed responded well to treatment with penicillin. Since *Mycoplasma* spp. lack a cell wall and therefore are naturally resistant to penicillin [7] it was assumed that pigs initially infected with *M. hyopneumoniae* had been secondarily superinfected with *Pasteurella multocida*. That assumption explained both the severity of the disease, and the positive effect of the treatment with penicillin during the early course of infection. That assumption was also supported by the fact that *P. multocida* was demonstrated in the lungs of both pigs, as was *M. hyopneumoniae* and *M. hyorhinis*.

However, the necropsy also identified the porcine lungworm *Metastrongylus apri* in the lungs of the affected pigs. Since lungworms had not been demonstrated at all in domestic pigs in Sweden for decades, their impact on the disease outbreak caused by *M. hyopneumoniae* and subsequently infected with *P. multocida* at the pasture was discussed.

**Fig. 1:**
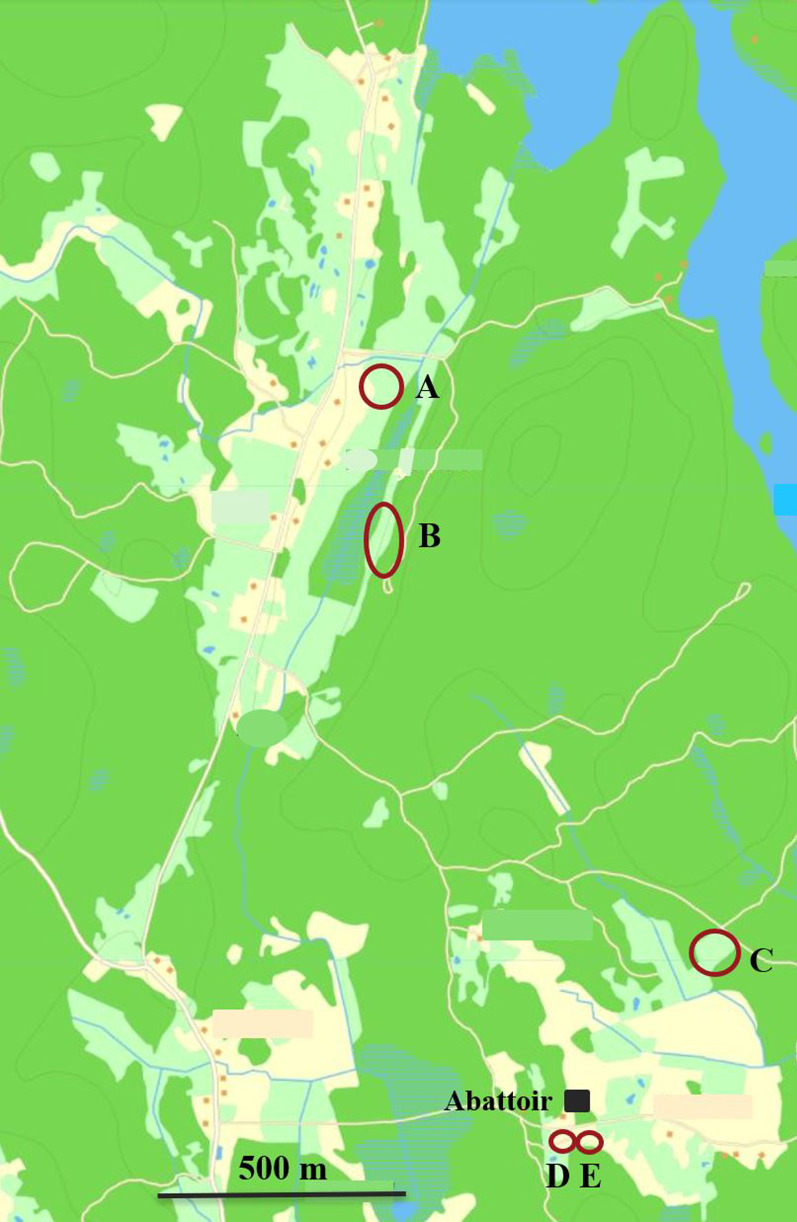
Geographic overview of the pastures and the flow of animals. Dark green areas represent forests; light green areas represent pastures; Yellow areas represent cultivated land; blue areas represent water. At the age of around 11 weeks, growers were transferred from the indoor facilities to pasture A, B or C. Approximately one month before reaching market weight they were transferred to either outdoor concrete slab D or E located close to the battoir

The affected farm had two other pastures, both located more than 300 m away from the affected pasture (Fig. 1), but none of them had been affected by respiratory disease. The pasture that had been affected by respiratory disease was geographically the most remote pasture, and wild boars were common in the neighbourhood. Lungworms are globally common in wild boars [8–10], including Sweden [11].

Due to the rare finding of lungworms, an intensified investigation with the aim of scrutinising the true impact of lungworms on the clinical course of the respiratory disease at the affected pasture was initiated.

## Results

### Clinical signs

Among the 131 fatteners that were allocated to pasture B, one pig was found dead four weeks after arrival, and occasionally dead pigs were found during the following week. Thereafter, the incidence of pigs affected by respiratory disease increased dramatically. In total, 30 of the 131 pigs (23%) were parenterally medicated with penicillin during the subsequent two weeks. The treatment efficacy was considered good when treatment was initiated during the early course of disease, but seven of the 30 treated pigs (23%) died. In total, another eight pigs either died or were euthanised without initiating treatment, whereof two were necropsied (see below). To conclude, 15 out of 131 pigs (11%) died during a period of three weeks. Pigs affected by disease searched shelter in the huts. As the weather was warm and dry, the huts were removed with the aim to prevent spread of disease, and thereafter the transmission of respiratory diseases decreased.

No similar signs of disease were recorded in other parts of the farm, neither on the other two pastures (Fig. 1; A and C) nor on the two concrete slabs (Fig. 1; D and E).

### Necropsies

Pneumonic lesions resembling those caused by *M. hyopneumoniae* were seen in the two pigs from pasture B that had been sent for necropsy. *M. hyopneumoniae*, *M. hyorhinis* and *P. multocida* were demonstrated in the lungs. Lungworms were found in the airways of both pigs, and these were identified as *Metastrongylus apri.*

### Parasitological investigations from faecal samples collected in the herd

No parasite eggs were detected in the faecal samples from growers aged ten weeks (n = 8) and not yet transferred to the pastures*, *i.e., when still at the indoor facilities (Table 1). Nor were any parasite eggs detected in pregnant sows that also were housed indoors (n = 8). None of these animals had been treated with anthelmintic drugs before sampling.

**Table 1:** Presence of parasite eggs at the outdoor facilities during the outbreak of severe respiratory disease at pasture B, as well in the indoor facilities in a subsequent investigation

Category	Indoor facilities		Outdoor facilities			
	Sows	Growers	Pasture C	Pasture B	Pasture A	Slab D
Age of pigs	Adults	10 weeks	4 months	5 months	6 months	7 months
Health status	Healthy	Healthy	Healthy	Respiratory disease	Healthy	Healthy
***Ascaris suum***						
Positive samples, n	0/8	0/8	0/3	4/5	0/5	3/4
Positive samples, prevalence	0%	0%	0%	80%	0%	75%
Epg, Mean ± SD	0	0	0	2090 ± 1545	0	1525 ± 2205
Epg, range	–	–	–	0–3800	–	0–4800
***Trichuris suis***						
Positive samples, n	0/8	0/8	0/3	4/5	0/5	0/4
Positive samples, prevalence	0%	0%	0%	80%	0%	0%
Epg, Mean ± SD	0	0	0	350 ± 453	0	0
Epg, range	–	–	–	0–1100	–	–
***Metastrongylus*** species						
Positive samples, n	0/8	0/8	0/3	1/5	1/5	1/4
Positive samples, prevalence	0%	0%	0%	20%	20%	25%
Epg, Mean ± SD	0	0	0	10 ± 23	10 ± 23	38 ± 75
Epg, range	–	–	–	0–50	0–50	0–150

During the outbreak of severe respiratory disease on pasture B, faecal samples from outdoor fatteners were collected from all pastures and concrete slabs that housed pigs (Table 1). In pigs aged five months at pasture B with respiratory disease, the mean faecal egg counts (FEC) for *A. suum* and *Trichuris suis* were 2090 ± 1545 (Max = 3800) egg per gram (Epg) and 35- ± 453 (Max 1100) Epg, respectively. In contrast, no eggs of these parasites were detected in faecal samples collected from pigs aged six months grazing on pasture A, nor from pigs aged four months and grazing on pasture C. Still, eggs of *A. suum* were detected in pigs aged seven months on the concrete slab D that had been populated with pigs from pasture A with a mean level of 2205 ± 1525 (Max 4800) Epg. Eggs of *Metastrongylus* spp. were demonstrated in the faeces from all pig categories aged 5 months or older (Table 1), but mean FEC was below 40 Epg in all groups.

### Results obtained at slaughter

**Fig. 2:**
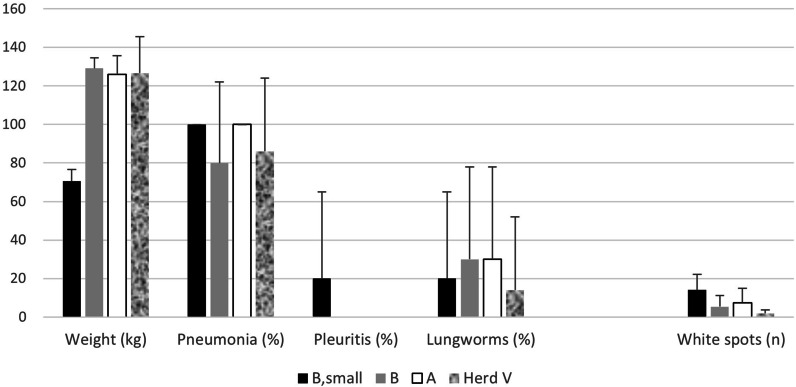
Mean values and standard deviations for live weights (kg), and incidences (%) of macroscopical lesions in the lungs at slaughter (healing mycoplasma-like pneumonias, pleuritis and presence of lungworms). Regarding white spots in livers, the mean number of white spots per pig is shown

The live weight corresponded to 129.2 ± 5.5 kg for the ten pigs that had reached market weight from pasture B, and 126.1 ± 9.7 kg for the ten market weight pigs from pasture A. The live weight for the five small pigs from pasture B was 70.5 ± 6.1 kg, which differed significantly (p < 0.001) from the pigs that had reached market weight (Fig. 2).

At the day of slaughter, also pigs from another pig herd (Herd V) that reared pigs to market weight outdoors were slaughtered. Herd V was situated 150 km from the affected herd and had not been diagnosed with respiratory diseases. The live weight of the seven pigs from herd V was 126.7 ± 18.8 kg (Fig. 2).

Most pigs had pneumonic lesions resembling mycoplasmosis, but these lesions were without exception in healing phases [12], i.e., none of them were actively in progress at the time of slaughter. Thereby they were not recorded by the official meat inspection at slaughter, which only register active processes [12]. Pleuritis was registered in one of the small pigs from pasture B, but not in any of the other pigs. The number of white spot liver lesions were somewhat higher among the small pigs from pasture B, but white spots were present in all categories of pigs. Also, adult lungworms were found in all categories of pigs on both farms (Fig. 2).

### Haematology at slaughter

**Fig. 3:**
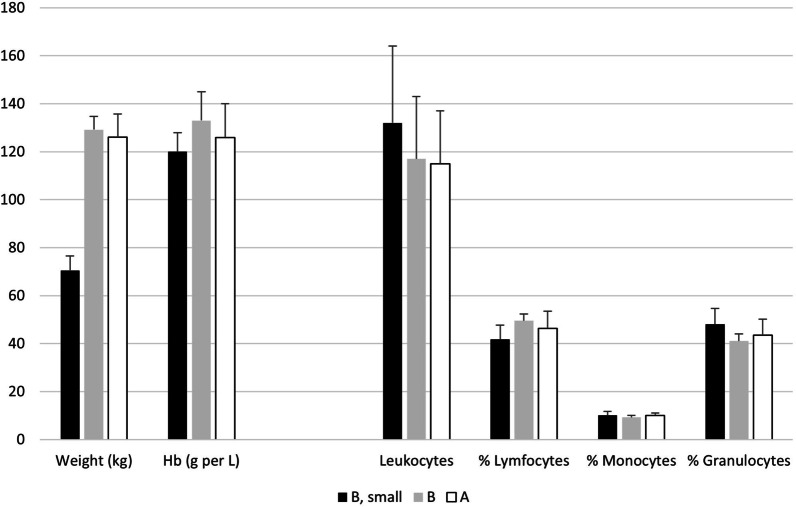
Mean values and standard deviations for live weights (kg), haemoglobin (Hb; gram per L) and total number of leukocytes (10^8^ per L). The figure also shows the subpopulations of the leukocytes as percentages of the total number of leukocytes

As seen in Fig. 3, the small pigs from pasture B had numerically higher leukocytes concentrations than the heavier pigs from pasture B and the healthy pigs from pasture A. However, these differences were not significant (p > 0.05). The small pigs from pasture B also had a significantly (p < 0.05) lower percentage of lymphocytes and somewhat (p = 0.07) higher percentage of granulocytes than the larger pigs from the same pasture, which altogether indicated a higher activation due to infections compared with the haematology of the larger pigs. All three categories had normal levels of haemoglobin.

### Serological reactions at slaughter

**Fig. 4:**
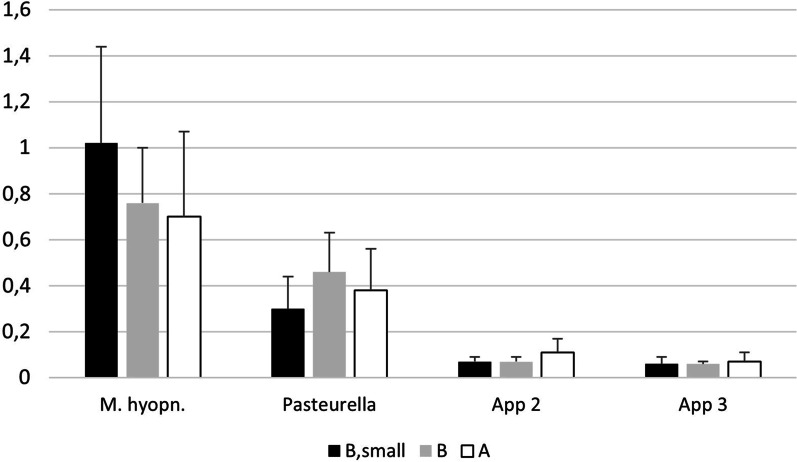
Absorbance levels (mean + standard deviations) of serum antibodies to Mycoplasma hyopneumoniae, Pasteurella multocida and Actinobacillus pleuropneumoniae serotypes 2 and 3. The cut off-value for positive reactions was 0.5 for all tests

All three categories of pigs were clearly seropositive to *M. hyopneumoniae*, and the absorbance levels were numerically but not significantly (p > 0.05) higher in the small pigs from pasture B than in large pigs There were seroreactors to *P. multocida* in all categories of pigs, but the mean absorbance levels were moderate and did not differ (p > 0.05) between pig categories. All pigs were seronegative to *Actinobacillus pleuropneumoniae* serotypes 2 and 3 (Fig. 4).

### Parasitological findings in faeces collected at slaughter

**Fig. 5:**
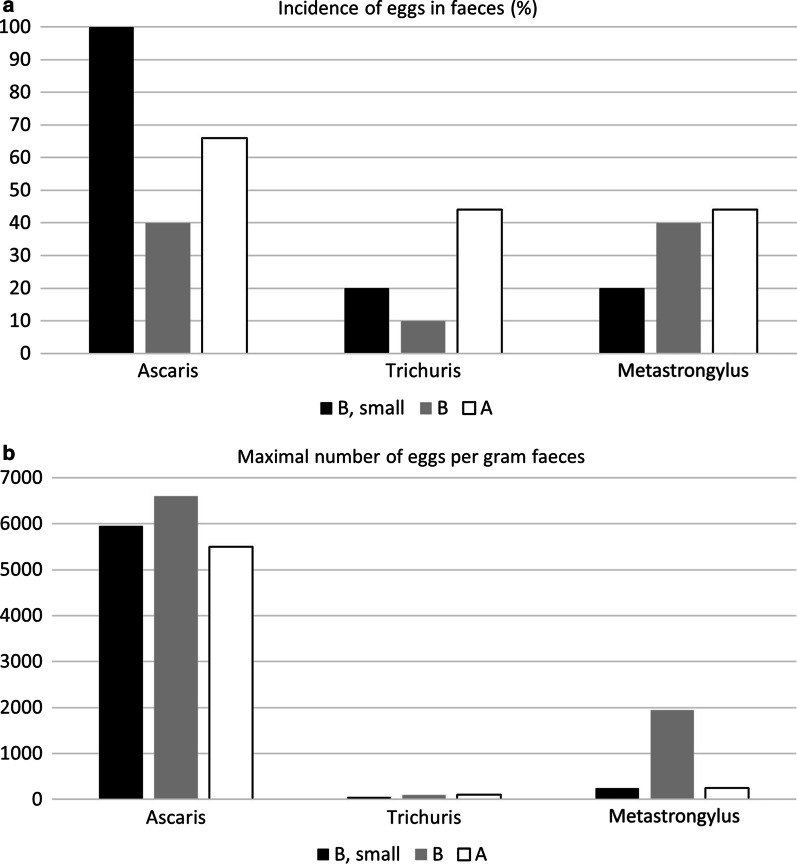
Occurrence of parasite eggs in feaces colleted at an individual level at slaughter (**a**), and the maximal number of eggs detected per gram faeces in an individual pig (**b**)

The 25 pigs were also examined for the presence of nematode eggs in faecal samples at an individual level. With the exception of *A. suum* where eggs were detected in all small pigs from pasture B (n = 5), the parasitological findings were similar in all three categories of pigs (Fig. 5).

Eggs of *Metastrongylus* spp., *T. suis* and *A. suum* were found in all categories of pigs: The mean levels of Epg were less than 300 and less than 30 for *Metastrongylus* spp. and *T. suis*, respectively. With respect to *A. suum*, the mean levels of Epg exceeded 1300 in all categories of pigs and exceeded 5500 Epg in individual pigs in each category (Fig. 5).

## Discussion

The porcine respiratory disease complex (PRDC) includes a number of pathogenic microbes and is dominated by bacterial species such as *Mycoplasma hyopneumoniae*, *Actinobacillus pleuropneumoniae* and *Pasteurella multocida* [13]. Respiratory parasites or parasites that include the respiratory system in their lifecycle, such as *A. suum* and *Metastrongylus* spp., may also have an impact on the PRDC. As lungworms are rarely detected and rarely discussed in the pig production today [6], a recent finding of lungworms in an outdoor pig herd, that also suffered a severe outbreak of respiratory disease, initiated this extended disease investigation. What was the true impact of the lungworms?

Growers that were transferred to a pasture when they were aged 10–11 weeks neither shed eggs of *A. suum* nor *Metastrongylus* spp. at that time. However, during the acute outbreak of respiratory disease in five-month-old pigs at pasture B, faecal analysis showed a low FEC of *Metastrongylus* spp. and a high FEC of *A. suum*. Both these parasites can contribute to respiratory disorders, *Metastrongylus* spp. by adult worms in the airways and *A. suum* because the parasite life cycle includes migration of larvae through the lungs [1].

It should however be noted that a low FEC of *Metastrongylus* spp. also was demonstrated in faecal samples from older outdoor pigs that did not show any clinical signs of respiratory disease, i.e. from six-month-old pigs at pasture A, as well as from seven-month-old pigs at the concrete slab D. In contrast, no eggs of *A. suum* were demonstrated in the pigs at pasture A, but a high FEC was demonstrated in pigs from that pasture that had been transferred to the concrete slab D prior to slaughter. This implied that pigs had been negative to *A. suum* at pasture A but became infected with a residual infection when transferred to the concrete slab prior to slaughter.

The results from the extended survey of lungs at slaughter showed to a large extent the same picture. The occurrence of mature lungworms in the airways of pigs from pasture B did not differ between small and large pigs, and adult lungworms were demonstrated in the airways of all groups of pigs that had been reared outdoors, i.e., not only from pigs reared at pasture B were severe respiratory diseases had been diagnosed. Adult lungworms were also demonstrated in the airways of the pigs from pasture A that apparently had been healthy during the rearing period—as well as in lungs of apparently healthy pigs from herd V that reared pigs outdoors 150 km away from the affected herd. The lungworms were not identified to species as our aim was to scrutinize the herd were *M. apri* already had been identified. Herd V was coincidentally slaughtering pigs on the same day and included in the study because they also reared pigs outdoors. Identifying species would have been interesting since herd V was located 150 km away and species within the *Metastrongylus* family may differ between geographically isolated wild boar populations [10]. The severity of the disease outbreak at pasture B was underlined by the high mortality and the weight difference of 50 kg between small and market weight pigs, which suggested a prolonged rearing time with around two months for the small pigs to reach desired market weight. In comparison with the market weight pigs from both pastures B and A, the small pigs from pasture B had a higher incidence of pigs with more than 20 white spots in the liver that presumably were caused by *A. suum* [1]. The small pigs also had numerically higher amounts of antibodies to *M. hyopneumoniae* than the heavier pigs. In addition, the small pigs from pasture B also had higher levels of leukocytes with lower percentages of lymphocytes but higher percentages of granulocytes than the heavier pigs, signs that indicated an ongoing response to infections. By merging these observations, the poor growth of the small pigs could be suggested to have been caused by infections that to some extent lasted throughout the whole rearing period [14].

Altogether, the recordings at slaughter and the necropsies strengthened the conclusion that the respiratory outbreak to a large extent was caused by *M. hyopneumoniae*. However, as affected pigs responded well to penicillin that does not combat *Mycoplasma* spp. [7] *P. multocida* was probably an important secondary pathogen in pigs that developed severe clinical signs during the disease outbreak. On the other hand, as the levels of antibodies to *P. multocida* were low in these pigs that had survived the acute disease outbreak, the long-term effect of *P. multocida* on surviving pigs appeared to have been marginal. This could probably be explained by the combination of initiating treatments early during the course of the infection and the removal of the huts where affected pigs searched shelter. As the huts had limited air space, they were thought to condense the pathogen load and, as the weather conditions allowed it, they were removed.

Considering the high FEC of *A. suum* in the five-month-old pigs at pasture B during the acute disease outbreak, it cannot be ruled out that *A. suum* contributed to the disease. The larvae of *A. suum* migrate through liver and lungs before maturation into adults [1] and thereby induce lesions in the lungs that may facilitates infections with e.g.* M. hyopneumoniae* and/or *P. multocida*. The suspicion of a negative impact of *A. suum* was further supported by the fact that infections with *A. suum* has been proven to reduce development of a protective immunity to *M. hyopneumoniae* following vaccination [15]. Indeed, it was notable that there were no signs at all of infections with *A. suum* in the older pigs at pasture A that had remained healthy during the time of the disease outbreak in pasture B. So why did these pigs not get sick when moved to the concrete slabs where they apparently became infected with *A. suum*? The explanation for this certainly is to find in the fact that the pigs at the time for transfer to the concrete slab since long had been exposed to *M. hyopneumoniae*. Thereby they had developed both antibodies and immunity towards (re)infections with *M. hyopneumoniae* at the time point when the larvae of *A. suum* migrated through their lungs [16, 17].

Taken together, the true impact of *Metastrongylus* spp. on the severe outbreak of respiratory disease in July 2021 was concluded to be negligible. The amount of *Metastrongylus* spp. eggs in the faecal samples collected at slaughter from *A. suum*-infected pigs that had grazed at pasture B was low and interestingly an antagonistic interaction between *A. suum* and *M. apri* has previously been demonstrated [18]. However, the levels of *Metastrongylus* spp. eggs found in the healthy and *A. suum*-negative pigs from pasture A were comparable with the levels found in *A. suum*-infected pigs affected by respiratory diseases in pasture B. In addition, adult lungworms were found in the lungs of 20–30% of the pigs from both pastures, and lungworms were also detected in another farm rearing pigs outdoors that was located 150 km away and not had reported any problems with respiratory diseases. The common observation of adult lungworms was somewhat surprising, but it must be emphasised that the routine inspections made at slaughter does not include inspection of the airways by opening them [19], and therefore the presence of adult lungworms in the airways of pigs will escape detection at routine meat inspections.

By opening the airways of the lungs in the extended inspection we discovered adult lungworms, but it is notable that the incidence of lungworms probably was underdiagnosed despite the extended measures undertaken. Eggs of *Metastrongylus* spp. were demonstrated in a total of nine of the 25 pigs from the affected herd, but adult worms were only demonstrated in the lungs in three of these pigs.

Looking at this the other way around; we found adult lungworms in a total of seven pigs, but we were only able to detect eggs of *Metastrongylus* spp. in the faeces from three of them. This could be due to factors such as adult worms not yet producing eggs or only adults of a single sex being present, preventing reproduction [10]. However, the low egg output also made underdiagnosing possible as the lower detection limit of the modified McMaster method used was 50 Epg. A total worm count and sex differentiation of the adult worms could have assisted in making further conclusions.

Despite extended efforts there is obviously a risk that *Metastrongylus* spp. remain undetected in individual pigs, but regardless of these diagnostic shortcomings we managed to demonstrate adult lungworms, or eggs in 13 out of 25 (52%) examined pigs from the affected herd at slaughter of which at least 10 pigs had not been associated with respiratory disease at all.

Seen from this perspective, and by also including the demonstration of adult lungworms in the other outdoor herd located 150 km away, it appeared reasonable to assume that *Metastrongylus* spp. also is present in other herds rearing pigs in outdoor systems, especially in areas with high densities of wild boars. The density of wild boars has been shown to be proportional to the presence of *Metastrongylus* spp. in earthworms [20], and to the parasitic burden in the wild boars themselves [21]. For this reason, parasites that can affect both wild boars and domestic pigs will be accumulated around feeding spots for wild boars [21], and it can be concluded that it must be inappropriate to establish feeding spots for wild boars in the vicinity of pig herds. Especially pig herds with access to outdoor grazing since wild boars actively visit pastures in search for food, facilitating a possible spread of infections. If wild boars are infected with *Metastrongylus* spp., which appears likely [8–11, 22], the soil of the pastures may become contaminated, and domestic pigs may consequently become infected via earthworms [1].

The wild boar population in Sweden has been evolving for decades and lungworms are frequently detected in animals younger than one year, but less frequently in older animals [11]. This is in accordance with observations from Corsica [22], Spain [8] and Italy [9], and it has been assumed that wild boars older than one year develop an immunity to the parasite.

The observations made in his study indicated that the burden of *Metastrongylus* spp. was low to moderate in the examined pig herds and the clinical impact of lungworms was therefore suggested to be limited. The possibility of *Metastrongylus* spp. predisposing to other infections should however be considered [23, 24].

The results obtained at the farrowing site located indoors revealed that the parasitic burden there was low to absent. Consequently, the presence of both *A. suum* and *Metastrongylus* spp. was related to the pastures and not to the growers that populated the pastures. Due to the high load of *A. suum* demonstrated in pasture B during the disease outbreak, it was decided to plough the pasture and grow crops for the next season, instead of grazing pigs. The long-term effect of such measure could of course be discussed as eggs of *A. suum*, may survive for years or even decades in the environment [25]. However, by rotating pastures the parasitic burden could hopefully be kept at moderate level.

The high number of *A. suum* eggs detected in samples from animals housed on concrete slabs was explained by the fact that pigs, if not already infected with *A. suum* on arrival, became infected by residual *A. suum* eggs. Residual eggs could be reduced by improving hygienic measures between batches [6].

The high burden of *A. suum,* in combination with the potential correlation between that parasite and the severe outbreak of respiratory disease at pasture B, highlighted that rotation of pastures ought to be considered when rearing pigs outdoors. Due to the high survivability of *A. suum eggs*, the best option would of course be to only use a pasture for one year in combination with growing crops for as many years as possible before again grazing pigs. However, in reality the access to land, labour and the extent of the production will decide the turn-over time for pasture rotation. On the other hand, the economic losses for disease outbreaks like the one described in pasture B will be significant [14], and subsequently the costs for improved measures to prevent such outbreaks ought to be regarded as profitable.

## Conclusion

As *Metastrongylus* spp. were demonstrated regardless of health status, and also in another healthy outdoor herd, the impact of *Metastrongylus* spp. on the outbreak of respiratory disease was depreciated. Instead, *Metastrongylus* spp. were suggested to be common in outdoor production, although rarely diagnosed. The reason for this is that *Metastrongylus* spp will escape detection at routine inspections at slaughter, and that they appeared to rarely induce clinical signs of respiratory disease. Instead, a possible association with a high burden of *A. suum* was suggested to have preceded the severe respiratory disease in pasture B.

## Materials and methods

### Herd

The affected herd was an integrated herd with 88 sows, were eight sows farrowed indoors every other week in an “age-segregated all in-all out system”. The offspring were reared indoors until the age of around eleven weeks when they were transferred to one out of three outdoor pastures (Fig. 1; A, B or C). The pastures were equipped with huts where pigs could seek shelter from harsh weather. Around one month before reaching a market weight of approximately 130 kg live weight, the pigs were transferred to concrete slabs located in absolute vicinity to the abattoir (Fig. 1, D or E). These concrete slabs were also located outdoors. Pigs on the slabs had access to water ponds and could seek shelter from harsh weather in the surrounding building.

### Clinical signs initiating the study

In May 2021, 131 pigs with a mean weight of approximately 30 kg were transferred to pasture B. A severe outbreak of respiratory disease took place in this group 4–7 weeks after the transfer. Two pigs that died were sent for necropsy at the National Veterinary Institute SVA, and the disease outbreak was further documented by looking into the records and treatment journals of the herd.

There had been no clinical signs of respiratory disease observed at the other pastures (Fig. 1; A and C) or at the concrete slabs of the herd (Fig. 1; D and E).

### Parasitological examinations of the herd

Coinciding in time with the disease outbreak in pasture B, faecal samples were collected from growers on all pastures (Fig. 1; A, B and C), as well as from one of the concrete slabs (D) that housed pigs close to market weight and that previously had been grazing at pasture A.

To obtain information regarding the parasite status of sows and of the offspring when transferred from the indoor facilities to the pastures, individual faecal samples were collected from one group of pregnant sows (n = 8) and pen samples were collected from eight different pens housing growers aged 10 weeks. None of these animals had been treated with anthelmintic drugs.

All faecal samples were analysed for the presence of parasite eggs with a centrifugal flotation technique. Nematode eggs were identified and quantified by a modified McMaster technique with a lower detection limit of 50 Epg faeces [26].

### Examinations made at slaughter

When the first pigs in the batch affected by the severe respiratory disease outbreak on pasture B reached market weight, ten market weight pigs and five of the smallest pigs from that batch were slaughtered and examined macroscopically with focus on presence of adult parasites in the airways. Presence of lungworms was denoted as positive or negative on site, and the lungworms were not defined with respect to species. As a control, an additional ten pigs that had been grazing on pasture A and had not been affected by respiratory disease, were also slaughtered and examined in the same way.

During the same day, outdoor pigs from another herd (herd V), located 150 km away from the affected herd, were also slaughtered at the abattoir. The lungs from seven pigs from herd V were examined for the presence of adult lungworms.

The individual slaughter weights were recorded for all these animals, and the live weight was calculated as the slaughter weight multiplied with 1.34. The internal organs were inspected in detail, the number of assumed *A. suum*-induced white spot lesions on the livers were counted, and the lungs were carefully examined with respect to lesions and the presence of adult parasites.

In addition, individual faecal samples were collected from the 25 pigs in the affected herd that was examined (n = 10 + 5 that had been grazing on the affected pasture B and the 10 healthy pigs that had been grazing on pasture A). These samples were analysed microscopically with focus on parasite eggs in the faeces as described above.

Individual blood samples without additives were also collected from the 25 pigs from pasture B (n = 10 + 5) and pasture A (n = 10), Following centrifugation the serum was separated and stored at -18ºC until analysed for the presence of antibodies to respiratory pathogens with different ELISA systems; *M. hyopneumoniae* (IDEXX *M. hyo.* Ab test, IDEXX, Westbrook, USA) *A. pleuropneumoniae* serotype 2 and 3 [27], and *P. multocida* [28].

Individual blood samples were also collected with EDTA as additive. These samples were analysed with respect to concentrations of haemoglobin and leukocytes, and the differential counts of the subpopulations of leukocytes (Exigo, Boule Medical AB, Spånga, Sweden).

### Statistics

Measurements presented are, unless specified otherwise, presented as mean values with standard deviations. Statistical analyses regarding body weights, levels of antibodies and leukocytes were carried out using student t test.

## Data Availability

Not applicable.

## Abbreviations

*A. Pleuropneumonia*: *Actinobacillus pleuropneumoniae*
*A. suum*: *Ascaris suum*
Epg: Egg per gram of faeces
FEC: Faecal Egg Count
*M. hyopneumoniae*: *Mycoplasma hyopneumoniae*
*M. hyorhinis*: *Mycoplasma hyorhinis*
*P. multocida*: *Pasteurella multocida*
Spp.: Species in plural
